# Childhood Obesity and COVID-19 Lockdown: Remarks on Eating Habits of Patients Enrolled in a Food-Education Program

**DOI:** 10.3390/nu13020383

**Published:** 2021-01-26

**Authors:** Mirella Nicodemo, Maria Rita Spreghini, Melania Manco, Rita Wietrzykowska Sforza, Giuseppe Morino

**Affiliations:** 1 Food Education Unit, Department of Pediatrics, Bambino Gesù Children’s Hospital, IRCCS, 00165 Rome, Italy; mariarita.spreghini@opbg.net (M.R.S.); rita.wietrzykowska@opbg.net (R.W.S.); gstefano.morino@opbg.net (G.M.); 2 Research Area for Multifactorial Disease and Complex Phenotypes, Bambino Gesù Children’s Hospital, IRCCS, 00165 Rome, Italy; melania.manco@opbg.net

**Keywords:** COVID-19 lockdown, obesity, children, eating habits, lifestyle

## Abstract

Childhood obesity is a worldwide health emergency. In many cases, it is directly linked to inappropriate eating habits and a sedentary lifestyle. During lockdown aimed at containing the coronavirus disease (COVID-19) spread, children have been forced to stay at home. The present study aimed at investigating the lifestyles of outpatients (aged 5–17 years) with complicated obesity enrolled in the day-hospital food education program at the Children’s Hospital Bambino Gesù in Rome. A survey was performed based on a structured questionnaire, investigating dietary habits and lifestyles. The questionnaire answers were rated as “yes/no/sometimes” or “often/never/sometimes”. Eighty-eight families correctly completed the questionnaire between March and May 2020. The results highlighted that 85.2% (*N* = 75) of the patients ate breakfast regularly, and 64.3% (*N* = 72) consumed fruit as an afternoon snack. However, 21.6% (*N* = 19) did just “often” home workouts, and 50.0% (*N* = 44) reported an increase of feeling hungry with “sometimes” frequency. There is a significant relationship of feeling hungry with gender (*p* < 0.0001) and age (*p* = 0.048) and, also, between gender with having breakfast (*p* = 0.020) and cooking (*p* = 0.006). Living a healthy lifestyle during lockdown was difficult for the outpatients, mainly due to the increase in a sedentary lifestyle and the increase in feeling hungry, but some healthy eating habits were maintained, as advised during the food education program provided before lockdown.

## 1. Introduction

Childhood obesity is one of the most pressing worldwide health emergencies, with a continuously increasing number of cases with severe and complicated obesity. The Italian data from the “OKkio alla Salute” surveillance system [1] showed that 20.4% of children (aged between eight and nine years) are overweight, and 9.4% are obese, with a greater diffusion in Southern and Central Italy. Most of the clinical forms of childhood obesity are directly linked to unhealthy eating habits, such as skipping breakfast, low fruit and vegetable intakes, high added sugars and saturated fat intakes, and poor physical activity, sedentariness with enhanced sedentary screen hours, reduced sleeping hours and poor sleep quality [1]. During summer vacation, with the school activities break, these negative behaviours seem to worsen with (i) the interruption of sport activities and school physical education, (ii) increase of the sedentary behaviours, (iii) greater access of unhealthy snacks, (iv) changes of daily planning and sleep–wake rhythm and (v) substitution of meals in the canteen with home-cooked meals (not always well-balanced) [2]. Indeed, during summer vacation, school children commonly register a certain body weight gain [3,4].

Likewise, during the lockdown aimed at containing the coronavirus disease (COVID-19) spread, children were suddenly forced to stay at home and away from school routines, following online classes for many hours without playing recreational sports outside or meeting their peers [3,4,5].

Listening to bad news continuously during the lockdown and experiencing important changes to their daily lives generated anxiety and fear in children [6]. In young children and adolescents, the lockdown had a greater impact on their emotional development compared to that in grown-ups [7]. Home confinement was associated with anxiety, which was attributable to the disruption in their daily routine and education, physical activities and opportunities for socialization and, above all, in pre-existing difficult situations, such as in children and adolescents with special needs (i.e., autism, attention deficit hyperactivity disorder, cerebral palsy, a learning disability, developmental delays and other behavioural and emotional difficulties) or socially deprived children (poor families with low finances or victims of violence) [7]. Moreover, it is well-known that children of parents with anxiety disorders are more likely to develop an anxiety disorder, so in this difficult time, the situation worsened for children and adolescents of parents with anxiety disorders [8]. Anxiety and stress caused an inevitable change in dietary choices, because many people found comfort and consolation through food consumption [9], especially of palatable high-fat and energy-dense foods [10].

At the Children’s Hospital Bambino Gesù in Rome, a food education program is offered to patients with complicated obesity (i.e., children with obesity presenting with severe insulin resistance, dyslipidaemia, hepatic steatosis, metabolic syndrome, hypertension, etc.). This kind of multicomponent, behavioural modification program is the foundation of all treatments for child weight loss and aims at long-term weight loss maintenance [11]. The interventions of the program include an interdisciplinary team of experts (paediatricians, registered dietitians and nurses). The food education program provides, first, a clinical evaluation and laboratory tests (nutritional assessment, blood chemistry, ultrasounds and specialist consultations). After, the patient is enrolled in an intensive lifestyle intervention program consisting of four monthly meetings. This program of food education includes an individual meeting with a dietitian and group educational therapy. During the individual meeting, the dietitian analyses the child’s seven-day food diary. This latter can be chosen by the child through a personal notebook where information about food consumption is collected. Specifically, the food diary can be a very useful tool for obtaining answers to specific questions (such as “What are you eating?”, “How much are you eating?”, “When are you eating?”, “Where are you eating?”, “How much are you drinking?”, “Do you play sports?”). Since the objective of the program is to educate on a healthy diet, the child and his family do not receive a detailed food plan but healthy food advice, according to the Mediterranean diet (e.g., with respect to the five meals; consumption of fruit as a midmorning snack and afternoon snack; consumption of vegetables at lunch and dinner; drink water instead of sugar-added drinks; consumption of a healthy dish with a first course based on pasta, rice, bread or cereals; a second course based on meat, legumes, fish, egg or dairy and a vegetable side dish; with respect to the macronutrient distribution in the main meals as breakfast, lunch and dinner and variations in food intakes); personalised advice on meal planning and food shopping and behaviour recommendations (i.e., at least 60 min of physical activity daily). An important aspect is the personalisation of food advice in order to meet the needs of the child and family, taking into account the lifestyle, the personal tastes, the socioeconomic factors and the cultural background. About nutrition, the dietitian provides information about portion control with no indication of kilocalories or grams of food consumption. The goal of this approach is to decrease the energy intake while improving the food quality. Therefore, at each meeting, the dietitian gives nutritional advice and makes changes based on the food diary and on the food behaviours of the child and his family. In this way, the child and family understand how to organise their weekly meal planning without detailed prescriptions. At the end of the day, a group meeting with children and parents is organised by the paediatrician and the dietitian, in which issues related to food education and a healthy lifestyle (e.g., food shopping, food choices, food labels, screen time, hydration and sleep quality) are discussed with the active involvement of everyone. This food education program is organised on a daily semi-hospitalised treatment every month, for up to four months.

The objective of our study was to investigate the eating habits and lifestyles during the COVID-19 lockdown in a population of children and adolescents (aged between five and 17 years) engaged in the food education program at the Children’s Hospital Bambino Gesù.

## 2. Materials and Methods

**Figure 1:**
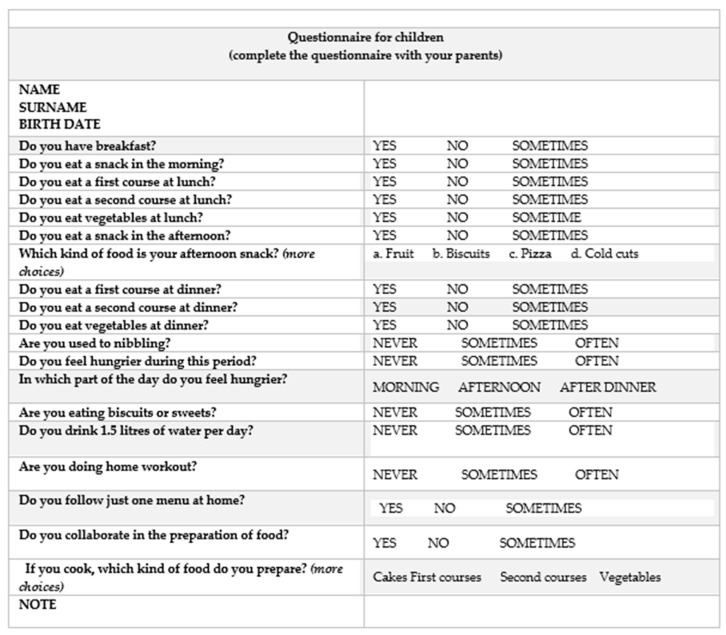
Description of the structured questionnaire used in the present study.

To explore the effects of a nationwide quarantine on a population of patients in a food education program, a structured nonvalidated questionnaire (Figure 1) was emailed to the parents on 31 March 2020. The emails were acquired from the patient’s electronic medical records.

The study was conducted in full agreement with the national and international regulations and the Declaration of Helsinki of 1975, revised in 2013. All parents were informed about the study requirements and were requested to accept the data sharing and privacy policy. About that, written informed consent was sent via email.

The inclusion criteria for the study were outpatients with obesity enrolled in the food education program from two months before lockdown, aged between 5 and 17 years, female and male genders. The patient had to fill in the questionnaire with a parent. The questionnaire consisted of 19 items regarding eating breakfast, the presence of two snacks in the day, healthy meals at lunch and dinner, drinking water, doing home workouts (11 questions), (ii) eating behaviour changes during the lockdown time (i.e., increase of feeling hungry and increase in the consumption of sugary foods) (4 questions), (iii) two specific questions on food choices (for snacks and homemade meals) and (iv) two specific questions on eating behaviours in the family (i.e., sharing a unique menu and cooking food together). The answers to the questions were defined according to a multichoice criterion:

- “yes/no/sometimes” for 11 questions
- “often/never/sometimes” for 5 questions
- “morning/afternoon/after dinner” for one question
- types of food for 2 questions
- along with the email, families received instructions to fill in the questionnaire
- the questionnaire was to be completed by the child with the supervision of the parents
- child personal details must be entered
- only one answer was acceptable for questions with “yes/no/sometimes” or “never/sometimes/often” options
- it was specified when more answers were possible for two questions
- please send the questionnaire by replying to this email address

The data collected with the questionnaire were analysed by extrapolating the frequency of each item considered in the proposed survey. Descriptive data was represented by the percentage. After, a cross-correlation among the different items was performed in order to underline specific relationships among them (e.g., consumption of snacks correlated to feeling hungry). For the association of different items of the questionnaire with gender and age, a chi-square test at *p*<0.05 was performed by XLSTAT (Addinsoft SARL, Paris, France).

## 3. Results

The email was sent to 100 families on 31 March 2020, and *N* = 95 (95%) of them replied by 31 May 2020. Seven questionnaires (7.4%) were excluded for the following reasons: six for incomplete data and one for double answers (by mother and son). A total of 88 questionnaires adequately compiled were received. The population (aged 11.8 with a standard deviation (SD) equal to 2.5) consisted of 32 male patients (36.4%) and 56 female patients (63.6%) with obesity (body mass index (BMI) 27.9 kg/m2 (SD 4.4)). The diagnosis of childhood obesity was based on the growth charts of the World Health Organization, 2007 (BMI > 97th percentile) [12]. Table 1 reports the answers of the questionnaires grouped according to the type of answer, and Table 2 reports the correlations of the different data.

From our survey, it appears that having breakfast is common in 85.2% of our population. While an equal distribution of eating a midmorning snack was highlighted (29.5% “yes”, 30.7% “no” and 39.8% “sometimes”), an afternoon snack was consumed in 81.8% of the population (Table 1). The first choice for an afternoon snack was fruit (64.3%) among biscuits, pizza and cold cuts (Table 1). Vegetable consumption was more common at dinner (69.3%) than at lunch (48.9%) (Table 1), so 46.6% of our population declared to eat vegetables in both meals (Table 2). In compliance with the dietary guidelines, 72.7% of the population did not eat a first course at dinner. The dietary guidelines provide for the preferable consumption of a first course at lunch; indeed, 71.6% of children ate a first course at lunch. Instead, it was less frequent to eat a second course at lunch (46.6%) than at dinner (86.4%) (Table 1). One of the most important aspects of the food education program is to share the same menu in the family: during lockdown, 55.7% of the population ate the same food in the family (Table 1). In order to involve children and adolescents and to bring them closer to healthy nutrition, the dietitian advises to cook food with their parents. During lockdown, the best way to kill time seemed to be cooking food; the survey highlighted that 51.1% of young patients took part in culinary preparations (Table 1), particularly in first courses (36.4%) and dessert recipes (26.5%) (Table 1). Furthermore, the population analysed reported an increase of feeling hungry (50.0% “sometimes”) (Table 1), especially in the afternoon (70.5%) (Table 1), and a related increase in the consumption of sweets and biscuits (72.7% “sometimes”) (Table 1). In our study, just 21.6% of the population “often” did home workouts during lockdown (Table 1). We also focused our attention on another eating behaviour that children generally neglected: drinking water. It was highlighted that just 46.6% of the population “often” drank 1.5 L of water per day (Table 1). Correlation of the data highlighted other aspects. In order to motivate an increase in feeling hungry and thinking that this behaviour was conditioned by the failure to respect the five meals, we did a correlation between the consumption of midmorning snacks and feeling hungry. Therefore, it was highlighted that 22.2% of children who do not consume a midmorning snack often felt hungry during the day, and just 11.5% of children who did consume a snack in the morning never felt hungry (Table 2). Furthermore, as expected, the population who did not have a snack in the morning was hungrier in the afternoon (77.8%) (Table 2). From evaluation of the data, it was also highlighted that, during lockdown, children who did not eat a midmorning snack nibbled sometimes (62.9%) or often (14.8%) (Table 2); quite the opposite, 34.6% of children used to eating a midmorning snack (Table 2) and 36% of children used to eating both snacks in the morning and afternoon (Table 2) did not nibble during the day. One of the most important aspects of the food education program is to consume for lunch a healthy and complete meal consisting of a first course, second course and vegetables. From the data analysis, it was shown that just 21.6% of the population (Table 2) had a complete lunch, but despite this, 68.4% of them felt hungry during the afternoon (Table 2).

The data were analysed in different gender and age groups. Table 3 shows the distribution of the population grouped by gender for the different items of the questionnaire. A chi-square test was used for the statistical analysis. Therefore, a significant relationship of gender was found with having breakfast (Χ^2^ = 5.419, *p* = 0.020), midmorning snack consumption (Χ^2^ = 7.246, 220 *p* = 0.027), frequency of nibbling (Χ^2^ = 6.457, *p* = 0.04), frequency of one family menu (Χ^2^ = 221 7.647, *p* = 0.0022) and frequency of cooking (Χ^2^ = 10.351, *p* = 0.006). Specifically, a significant relationship with feeling hungry in different moments of the day (Χ^2^ = 32.244, *p* < 0.0001) was highlighted; indeed, 71.4% of females and 71.9% of males felt hungrier in the afternoon, while 14.3% of females and 18.8% of males during the morning and 14.3% of females and 9.4% of males after dinner. Table 4 shows the distributions of the population grouped by age according to the different items of the questionnaire. A significant relationship of age with feeling hungry during the day (Χ^2^ = 6.053, *p* = 0.048) was found; indeed, 77.8% of group 1 (age ≤ 10 years) and 68.9% of group 2 (age ≥ 11 years) felt hungrier in the afternoon, while 22.2% of group 1% and 13.1% of group 2 during the morning and no one of group 1% and 18.0% of group 2 after dinner. No significant association of age with the other items was highlighted.

## 4. Discussion

The present study showed that, during the nationwide quarantine due to COVID-19, having a healthy lifestyle was difficult for children with obesity, despite the fact that the relevant population was taking part in a food education program in the hospital. Indeed, it is well-known that, at this time, obese patients were under a great deal of stress, which led them to a sedentary life and unhealthy food consumption [13].

The interruption of school and sport activities, the reorganization of the day for children (online lessons) and for adults (smart-working or no work), the deprivation of parental companionship (e.g., children quarantined at institutions or detached from parents due to quarantine) [14] and the bad moods induced by listening to sad news through the media every day likely had an effect on the increase of stress and anxiety. All of these conditions led people toward an increase in feeling hungry and a consequent increase of sugary and appetising food consumption [15] and toward an alarming decrease of physical activity. Children enrolled in the food education program also reported an increase of feeling hungry and a relevant increase in sweet consumptions. These results were also confirmed by the Sidor and Rzymski study on overweight, obese and older population (aged >18): 43.5% of cases declared an increase in the amount of food eaten during lockdown, and 51.8% admitted an increase in the number of snacks during the day between meals [16]. Evidently, the main problem for children and adolescents during lockdown was stress and anxiety from the unexpected and sudden changes in lifestyle. Many evidences show that stress, in addition to being an influence on children’s lifestyles, stimulates eating in the absence of hunger, especially sweet foods [17].

One of the most relevant issues for children was the decrease in physical activity and the increase in a sedentary lifestyle during the COVID-19 lockdown. This evidence is correlated with the wrong behaviour of Italian children with obesity spending three-to-four hours of the day in sedentary activities [1]. The alarming consequences of these behaviours are the risk of overweight/obesity and cardiovascular complications [18]. The same goes for adults: indeed, in another Italian observational study to evaluate the changes in weights and eating habits of adults with obesity treated in the Obesity Unit (“Città della Salute e della Scienza Hospital of Torino”), most individuals reduced their levels of physical activity from before quarantine (46.7%) or did not practice exercise during the lockdown (32.6%) [19]. Similar results were observed by a study on health-related behaviours among Spanish children and adolescents: a significant reduction of the weekly minutes of physical activity was highlighted during the COVID-19 confinement (−102.5, SD 159.6) [20].

Some healthy eating habits imparted during the food education program were preserved in most cases: having breakfast, eating fruit as an afternoon snack and vegetables at lunch and dinner.

An important outcome is the high percentage of children who had breakfast, unlike the eating habits of Italian children: the “OKkio alla Salute, 2019” data highlighted that 8.7% of children do not have breakfast and 35.6% of children eat an inadequate breakfast [1], and the “Health Behaviour in School-aged Children, 2018 (HBSC,2018)” data showed that the habit of not having breakfast is common in adolescents (20.7% at 11 years, 26.4% at 13 years and 30.6% at 15 years) [21]. Surely, due to the food advice, children enrolled in the food education program knew the importance of having breakfast, but above all, they had more time available during the lockdown than the school term.

It is worth noting that 64.3% of children chose fruits for snacks, and about fifty percent (46.6%) ate vegetables at lunch and dinner. These results showed better percentages than the Italian data of “Okkio alla Salute”, where 55.2% of children (aged eight and nine) had an oversized morning snack, and 24.3% ate fruits and/or vegetables less than once a day. A better situation was also shown with respect to the international data of “HBSC, 2018”, in which 35.4% of adolescents (aged 11–15) consumed at least one portion of fruit daily, and 27.3% ate vegetables at least once a day [21]. Therefore, children enrolled in the food education program were likely well-behaved and able to make healthy food choices. On the contrary, 18% of adults treated in the Obesity Unit of Hospital of Torino reported a decrease in fruits and vegetables consumption during the lockdown [19].

On the other hand, the increase of feeling hungry and of nibbling must be linked to the failure to respect the five meals and to an incomplete meal at lunch. These healthy habits were not fully acquired. Certainly, these inappropriate food behaviours are also related to this period’s difficulties and, especially, to the stress and anxiety from the unexpected and sudden changes of lifestyle. Indeed, during the COVID-19 lockdown, hunger perception also increased in adults during different moments of the day (before the main meals, between them and after dinner) [15].

The main limitation of the present study was that the population participated in only two of four meetings, and therefore, the food advice received was incomplete. Another limitation was represented by the lack of data on the socio-economic, cultural aspects and educational levels of the parents. Furthermore, no data available before the lockdown about the items of the questionnaire was a weak aspect of the study, preventing further analyses. In addition, the administration of a nonvalidated questionnaire was recognized as a limitation. However, a strong point was that we can monitor the lifestyle changes in this population over time with the same questionnaire.

## 5. Conclusions

In this study, we assessed the eating habits and lifestyles of paediatric outpatients with medically complicated obesity during the COVID-19 lockdown in Italy from 9 March 2020 to 18 May 2020. These results highlight clearly that more support must be provided to children with obesity in special situations that change their regular school life, such as during the COVID-19 lockdown but, also, during the summer or Christmas holidays. However, since 8 October 2020, the COVID-19 pandemic is forcing a second phase of lockdown, and long-lasting effects on the paediatric population should be studied in order to think about support strategies. Analysing the impact of lockdown on the lifestyle habits of the obese children of North, Central and South Italy could represent a first step, such as the Lost in Italy (Lockdown and lifeSTyles IN ITALY) project, which is evaluating the impact of COVID-19 on the physical, mental and social wellbeing of the elderly population (≥65 years) in Lombardy, the region most heavily affected by the pandemic [22].

## Figures and Tables

**Table 1:** Answers of questionnaires.

**Frequency (%) of Meals** **and Family Habits** **during Lockdown**						
			YES	NO		SOMETIMES
BREAKFAST (*)			75 (85.2%)	0 (0.0%)		13 (14.8%)
MIDMORNING SNACK (*)			26 (29.5%)	27 (30.7%)		35 (39.8%)
FIRST COURSE AT LUNCH (*)			63 (71.6%)	7 (8.0%)		18 (20.5%)
SECOND COURSE AT LUNCH (*)			41 (46.6%)	15 (17.0%)		32 (36.4%)
VEGETABLES AT LUNCH (*)			43 (48.9%)	13 (14.8%)		3 (36.4%)
AFTERNOON SNACK (*)			72 (81.8%)	1 (1.1%)		15 (17.0%)
FIRST COURSE AT DINNER (*)			5 (5.7%)	64 (72.7%)		19 (21.6%)
SECOND COURSE AT DINNER (*)			76 (86.4%)	2 (2.3%)		10 (11.4%)
VEGETABLES AT DINNER (*)			61 (69.3%)	4 (4.5%)		23 (26.1%)
FAMILY MENU (**)			49 (55.7%)	18 (20.5%)		21 (23.9%)
COOKING FOOD (**)			45 (51.1%)	15 (17.0%)		28 (31.8%)
**Choice of different food for afternoon snack during lockdown**						
	FRUIT	BISCUITS	PIZZA		COLD CUTS	
FOOD FOR AFTERNOON SNACK	72 (64.3%)	25 (22.3%)	4 (3.6%)		11 (9.8%)	
**Frequency (%) of eating, drinking and lifestyle behaviours during lockdown**						
			NEVER	SOMETIMES		OFTEN
NIBBLING (a)			26 (29.5%)	46 (52.3%)		16 (18.2%)
FEELING HUNGRY (a)			18 (20.5%)	44 (50.0%)		26 (29.5%)
EATING SWEETIES AND BISCUITS (a)			12 (13.6%)	64 (72.7%)		12 (13.6%)
DRINKING 1.5 LITERS OF WATER PER DAY (b)			16 (18.2%)	31 (35.2%)		41 (46.6%)
HOME WORKOUT (c)			27 (30.7%)	42 (47.7%)		19 (21.6%)
**Children (%) and feeling hungry in different moments of the day**						
			MORNING	AFTERNOON		AFTER DINNER
FEELING HUNGRY DURING THE DAY			14 (15.9%)	62 (70.5%)		12 (13.6%)
**Different kinds of food cooked by the children**						
	CAKES	FIRST COURSES	SECOND COURSES		VEGETABLES	
FOOD COOKED	35 (26.5%)	48 (36.4%)	26 (19.7%)		23 (17.4%)	

(*) Meals during lockdown. (**) Family Habits during lockdown. (a) Eating behaviour during lockdown. (b) Drinking behaviour during lockdown. (c) Lifestyle behaviour during lockdown.

**Table 2:** Correlation of data obtained from the questionnaires.

**Consumption of Midmorning Snack and Feeling Hungry**				
		Feeling hungry		
		NEVER	SOMETIMES	OFTEN
Consumption of midmorning snack	YES	3 (11.5%)	14 (53.9%)	9 (34.6%)
	NO	9 (33.3%)	12 (44.4%)	6 (22.2%)
	SOMETIMES	6 (17.1%)	18 (51.4%)	11 (31.4%)
**Consumption of midmorning snack and feeling hungry in a specific part of the day**				
		Feeling hungry in a specific part of the day		
		MORNING	AFTERNOON	AFTER DINNER
Consumption of midmorning snack	YES	4 (16.7%)	16 (66.6%)	4 (16.27%)
	NO	2 (7.4%)	21 (77.8%)	4 (14.8%)
	SOMETIMES	8 (22.8%)	25 (71.4%)	2 (5.7%)
**Consumption of midmorning snack and nibbling**				
		Nibbling		
		NEVER	SOMETIMES	OFTEN
Consumption of midmorning snack	YES	9 (34.6%)	8 (30.7%)	9 (34.6%)
	NO	6 (22.2%)	17 (62.9%)	4 (14.8%)
	SOMETIMES	11 (31.4%)	21 (60%)	3 (8.6%)
**Consumption of complete lunch**				
	YES			
First course, second course and vegetables	19 (21.6%)			
**Consumption of complete lunch and feeling hungry in a specific part of the day**				
		Feeling hungry in a specific part of the day		
		MORNING	AFTERNOON	AFTER DINNER
Consumption of complete lunch	YES	3 (15.8%)	13 (68.4%)	3 (15.8%)
**Consumption of midmorning snack and afternoon snack**				
	YES			
Midmorning snack and afternoon snack	25(28.4%)			
**Consumption of midmorning snack and afternoon snack and nibbling**				
		Nibbling		
		NEVER	SOMETIMES	OFTEN
Consumption of midmorning snack and afternoon snack	YES	9 (36.0%)	8 (32%)	8 (32%)
**Consumption of vegetables at lunch and dinner**				
	YES			
Vegetables at lunch and dinner	41 (46.6%)			

**Table 3:** Distribution of children by gender for the different items of the questionnaire.

	Group 1 Female (*N* = 56; 63.6%)	Group 2 Male (*N* = 32; 36.4%)	Chi-Square (*p*-Value)		Group 1 Female (*N* = 56; 63.6%)	Group 2 Male (*N* = 32; 36.4%)	Chi-Square (*p*-Value)
**Do you have breakfast?**				**Are you used to nibbling?**			
YES	44 (78.6%)	31 (96.9%)	**Χ^2^ = 5.419 (0.020)**	NEVER	13 (23.2%)	13 (40.6%)	**Χ^2^ = 6.457 (0.04)**
SOMETIMES	12 (21.4%)	1 (3.1%)		SOMETIMES	35 (62.5%)	11 (34.4%)	
NO	-	-		OFTEN	8 (14.3%)	8 (25.0%)	
**Do you eat a snack in the morning?**				**Do you feel hungrier during this period?**			
YES	15 (26.8%)	11 (34.4%)	**Χ^2^ = 7.246 (0.027)**	NEVER	11 (19.6%)	7 (21.9%)	**Χ^2^ = 0.499 (0.779)**
NO	13 (23.2%)	14 (43.8%)		SOMETIMES	27 (48.2%	17 (53.1%)	
SOMETIMES	28 (50.0%)	7 (21.9%)		OFTEN	18 (32.1%)	8 (25.0%)	
**Do you eat a first course at lunch?**				**In which part of the day do you feel hungrier?**			
YES	37 (66.1%)	26 (81.3%)	**Χ^2^ = 3.801 (0.150)**	MORNING	8 (14.3%)	6 (18.8%)	**Χ^2^ = 32.244 (<0.0001)**
NO	4 (7.1%)	3 (9.4%)		AFTERNOON	40 (71.4%)	23 (71.9%)	
SOMETIMES	15 (26.8%)	3 (9.4%)		AFTER DINNER	8 (14.3%)	3 (9.4%)	
**Do you eat a second course at lunch?**				**Are you eating biscuits or sweets?**			
YES	23 (48.1%)	18 (56.3%)	**Χ^2^ = 2.005 (0.367)**	NEVER	8 (14.3%)	4 (12.5%)	**Χ^2^ = 0.919 (0.632)**
NO	10 (17.9%)	5 (15.6%)		SOMETIMES	39 (69.6%)	25 (78.1%)	
SOMETIMES	23 (41.1%)	9 (28.1%)		OFTEN	9 (16.1%)	3 (9.4%)	
**Do you eat vegetables at lunch?**				**Do you drink 1.5 litres of water per day?**			
YES	30 (53.6%)	13 (40.6%)	**Χ^2^ = 1.488 (0.475)**	NEVER	11 (19.6%)	5 (15.6%)	**Χ^2^ = 3.438 (0.179)**
NO	7 (12.5%)	6 (18.8%)		SOMETIMES	23 (41.1%)	8 (25.0%)	
SOMETIMES	19 (33.9%)	13 (40.6%)		OFTEN	22 (39.3%)	19 59.4%)	
**Do you eat a snack in the afternoon?**				**Are you doing home workout?**			
YES	44 (78.6%)	28 (87.5%)	**Χ^2^ = 3.684 (0.158)**	NEVER	15 (26.8%)	12 (37.5%)	**Χ^2^ = 1.117 (0.572)**
NO	0 (0%)	1 (3.1%)		SOMETIMES	28 (50.0%)	14 (43.8%)	
SOMETIMES	12 (21.4%)	3 (9.4%)		OFTEN	13 (23.3%)	6 (18.8%)	
**Which kind of food is your afternoon-snack?**				**Do you follow just one menu at home?**			
FRUIT	45 (65.2%)	27 (62.8%)	**Χ^2^ = 0.545 (0.909)**	NEVER	25 (44.6%)	24 (75.0%)	**Χ^2^ = 7.647 (0.0022)**
BISCUITS	16 (23.2%)	9 (20.9%)		SOMETIMES	14 (25.0%)	4 (12.5%)	
PIZZA	2 (2.9%)	2 (4.7%)		OFTEN	17 (30.4%)	4 (12.5%)	
COLD CUTS	6 (8.7%)	5 (11.6%)					
**Do you eat a first course at dinner?**				**Do you collaborate in the preparation of food?**			
YES	3 (5.4%)	2 (6.3%)	**Χ^2^ = 0.252 (0.882)**	YES	35 (62.5%)	10 (31.5%)	**Χ^2^ = 10.351) (0.006)**
NO	40 (71.4%)	24 (75.0%)		NO	5 (8.9%)	10 (31.5%)	
SOMETIMES	13 (23.2%)	6 (18.8%)		SOMETIMES	16 (28.6%)	12 (37.5%)	
**Do you eat a second course at dinner?**				**If you cook, which kind of food do you prepare?**			
YES	49 (87.5%)	27 (84.4%)	**Χ^2^ = 3.698 (0.157)**	SWEETIES	25 (25.5%)	10 (29.4%)	**Χ^2^ = 2.323 (0.508)**
NO	-	2 (6.3%)		FIRST COURSE	33 (33.7%)	15 (44.1%)	
SOMETIMES	7 (12.5%)	3 (9.4%)		SECOND COURSE	21 (21.4%)	5 (14.7%)	
**Do you eat vegetables at dinner?**				VEGETABLES	19 (19.4%)	4 (16.7%)	
YES	41 (73.2%)	20 (62.5%)	**Χ^2^ = 1.162 (0.559)**				
NO	2 (3.6%)	2 (6.3%)					
SOMETIMES	13 (23.2%)	10 (31.3%)					

**Table 4:** Distribution of children by age for the different items of the questionnaire.

	Group 1 Age ≤ 10 Years(*N* = 27; 30.7%)	Group 2 Age ≥11 Years(*N* = 61; 69.3%)	Chi-Square (*p*-Value)		Group 1 Age ≤ 10 Years(*N* = 27; 30.7%)	Group 2 Age ≥ 11 Years(*N* = 61; 69.3%)	Chi-Square (*p*-Value)
**Do you have breakfast?**				**Are you used to nibbling?**			
YES	24 (88.9%)	51 (83.6%)	**Χ^2^ = 0.415 (0.520)**	NEVER	8 (29.6%)	18 (29.5%)	**Χ^2^ = 0.004 (0.998)**
SOMETIMES	0 (0%)	0 (0%)		SOMETIMES	14 (51.8%)	32 (52.4%)	
NO	3 (11.1%)	10 (16.4%)		OFTEN	5 (18.5%)	11 (18.0%)	
**Do you eat a snack in the morning?**				**Do you feel hungrier during this period?**			
YES	10 (37.0%)	16 (26.2%)	**Χ^2^ = 1.100 (0.577)**	NEVER	7 (25.9%)	11 (18.0%)	**Χ^2^ = 2.619 (0.270)**
NO	7 (25.9%)	20 (32.8%)		SOMETIMES	10 (37.0%)	34 (55.7%)	
SOMETIMES	10 (37.0%)	25 (40.9%)		OFTEN	10 (37.0%)	16 (26.2%)	
**Do you eat a first course at lunch?**				**In which part of the day do you feel hungrier?**			
YES	18 (66.7%)	45 (73.8%)	**Χ^2^ = 0.717 (0.699)**	MORNING	6 (22.2%)	8 (13.1%)	**Χ^2^ = 6.053 (0.048)**
NO	2 (7.4%)	5 (8.2%)		AFTERNOON	21 (77.8%)	42 (68.9%)	
SOMETIMES	7 (25.9%)	11 (18.0%)		AFTER DINNER	0 (0%)	11 (18.0%)	
**Do you eat a second course at lunch?**				**Are you eating biscuits or sweets?**			
YES	13 (48.2%)	28 (45.9%)	**Χ^2^ = 0.168 (0.919)**	NEVER	4 (14.8%)	8 (13.1%)	**Χ^2^ = 0.109 (0.974)**
NO	5 (18.5%)	10 (16.4%)		SOMETIMES	19 (70.4%)	45 (73.8%)	
SOMETIMES	9 (33.3%)	23 (37.7%)		OFTEN	4 (14.8%)	8 (13.1%)	
**Do you eat vegetables at lunch?**				**Do you drink 1.5 litres of water per day?**			
YES	12 (44.4%)	31 (50.8%)	**Χ^2^ = 3.074 (0.215)**	NEVER	6 (22.2%)	10 (16.4%)	**Χ^2^ = 0.716 (0.699)**
NO	2 (7.4%)	11 (18.0%)		SOMETIMES	8 (29.6%)	23 (37.7%)	
SOMETIMES	13 (48.1%)	19 (31.1%)		OFTEN	13 (48.1%)	28 (45.9%)	
**Do you eat a snack in the afternoon?**				**Are you doing home workout?**			
YES	25 (92.6%)	47 (77.0%)	**Χ^2^ = 3.118 (0.210)**	NEVER	7 (25.9%)	20 (32.8%)	**Χ^2^ = 4.163) (0.125)**
NO	0 (0%)	1 (1.6%)		SOMETIMES	17 (62.9%)	25 (40.9%)	
SOMETIMES	2 (7.4%)	13 (21.3%)		OFTEN	3 (11.1%)	16 (26.2%)	
**Which kind of food is your afternoon-snack?**				**Do you follow just one menu at home?**			
FRUIT	21 (63.6%)	51 (64.5%)	**Χ^2^ = 0.144 (0.986)**	NEVER	13 (48.1%)	36 (59.0%)	**Χ^2^ = 2.044 (0.360)**
BISCUITS	8 (24.2%)	17 (21.5%)		SOMETIMES	8 (29.6%)	10 (16.4%)	
PIZZA	1 (3.0%)	3 (3.8%)		OFTEN	6 (22.2%)	15 (24.6%)	
COLD CUTS	3 (9.1%)	8 (10.1%)					
**Do you eat a first course at dinner?**				**Do you collaborate in the preparation of food?**			
YES	3 (11.1%)	2 (3.8%)	**Χ^2^ = 2.870 (0.238)**	YES	15 (55.5%)	30 (49.2%)	**Χ^2^ = 0.321 (0.852)**
NO	17 (62.9%)	47 (77.0%)		NO	4 (14.8%)	11 (18.0%)	
SOMETIMES	7 (25.9%)	12 (19.7%)		SOMETIMES	8 (29.6%)	20 (32.8%)	
**Do you eat a second course at dinner?**				**If you cook, which kind of food do you prepare?**			
YES	20 (74.1%)	56 (91.8%)	**Χ^2^ 5.074 (0.079)**	SWEETIES	14 (31.8%)	21 (23.9%)	**Χ^2^ = 3.342 (0.342)**
NO	1 (3.7%)	1 (1.6%)		FIRST COURSE	12 (27.3%)	36 (40.9%)	
SOMETIMES	6 (22.2%)	4 (6.5%)		SECOND COURSE	8 (18.2%)	18 (20.4%)	
**Do you eat vegetables at dinner?**				VEGETABLES	10 (22.7%)	13 (14.8%)	
YES	17 (62.9%)	44 (72.1%)	**Χ^2^ = 1.060 (0.589)**				
NO	1 (3.7%)	3 (4.9%)					
SOMETIMES	9 (33.3%)	14 (22.9%)					

## Data Availability

All data analysed in this study are available upon request to the corresponding author.
